# Re-sequencing of 180 bitter gourd accessions uncovering the genetic basis of key horticultural traits

**DOI:** 10.3389/fpls.2026.1807943

**Published:** 2026-04-16

**Authors:** Chengcheng Feng, Xixi Ju, Xiongjuan Huang, Jiazuo Liang, Yuhui Huang, Xiaofeng Chen, Xinglian Liu, Rukui Huang, Wenjin Yu

**Affiliations:** 1 College of Agriculture, Guangxi University, Nanning, China; 2 Vegetable Research Institute, Guangxi Academy of Agricultural Sciences, Nanning, China

**Keywords:** Bitter gourd, genome-wide association studies, pleiotropic locus, population structure, re-sequencing

## Abstract

Bitter gourd (*Momordica charantia* L.) is a valuable cucurbit crop, yet its genetic improvement has been hindered by limited genomic resources and understanding of trait genetics. Here, we performed whole-genome re-sequencing of a diverse panel of 180 bitter gourd accessions, generating an average coverage of 33.91× and identifying ~1.78 million high-quality single nucleotide polymorphisms (SNPs). Population structure analysis delineated the panel of 180 bitter gourd accessions into five distinct sub-populations, revealing moderate genetic diversity and varied linkage disequilibrium decay patterns across subgroups. We phenotyped the panel for four key horticultural yield related traits and conducted genome-wide association studies (GWAS). This identified several significant loci, including a major pleiotropic region on chromosome MC04 concurrently associated with single fruit weight (SFW), fruit length (FL), and fruit diameter (FD). Haplotype analysis of candidate genes within this interval (MC04g0117, MC04g0120, MC04g0121 and MC04g0126) demonstrated that Hap1 significantly enhanced SFW (20.46%—22.68%), FL (13.68%—16.09%), and FD (6.28%—8.44%) compared to Hap2. Notably, although GWAS did not initially link this region to fruit flesh thickness (FFT), haplotype partitioning revealed that Hap2 consistently increased FFT by 4.00%—4.31%, uncovering a previously hidden phenotypic effect. Furthermore, trait-specific loci were mapped, such as for SFW on MC08 (MC08g1837) and FFT on MC09 (MC09g_new0383, MC09g1204), with corresponding haplotypes conferring significant phenotypic advantages. Our study provides a comprehensive genomic variation map for bitter gourd, elucidates the genetic architecture of crucial yield-related traits, and identifies candidate genes with clear haplotype effects, thereby establishing a foundational resource for accelerating molecular breeding programs.

## 1 Introduction

Bitter gourd (*Momordica charantia*, 2n = 2x = 22), a member of the *Cucurbitaceae* family, characterized by its spiny skin and distinctive bitter taste. Since bitter gourd is rich in vitamins, dietary fiber, amino acids, and bioactive compounds, it holds significant economic and medicinal value (Urasaki et al., 2017; Tan et al., 2016; Chan et al., 2020; Krawinkel and Keding, 2006; Farooqi et al., 2018; Dia and Krishnan, 2016; Lee-Huang et al., 1995). Bitter gourd, which originated in and was domesticated in Asia (Schaefer and Renner, 2010), is now widely cultivated and consumed across the continent, with its cultivation area expanding by approximately 340,000 hectares annually (Mccreight et al., 2013).

Currently, numerous studies have been conducted to explore the significant economic and medicinal value of bitter gourd. However, progress in uncovering the genetic basis of its horticulturally important traits has largely lagged behind that of food and oil crops, which is primarily due to the limited availability of genomic information and genetic resources. For example, commercial cultivars have a high degree of uniformity, while there is also low genetic diversity among available germplasm (Dhillon et al., 2016). To facilitate genetic mapping and breeding new cultivars, researches have significantly expanded genomic information of bitter gourd through assembling two scaffold-level genome of OHB3–1 and Dali-11, and a telomere-to-telomere (T2T) gap-free genome of *Momordica charantia* L. var. *abbreviata* Ser (Urasaki et al., 2017; Cui et al., 2020; Fu et al., 2023). These available genome assemblies have greatly facilitated extensive genetic analysis and QTL mapping of agronomic traits, such as fruit quality, yield, and disease resistance (Rao et al., 2021; Kaur et al., 2021a, Kaur et al., 2021b; Yang et al., 2022; Liu et al., 2024; Vinay et al., 2023; Zhong et al., 2023a, Zhong et al., 2023b). These studies, however, predominantly utilize artificial populations. For example Huang et al. (2024) used re-sequencing data from a RIL populatin consist of 300 lines to build a high-density genetic map and identified several loci for powdery mildew resistance through QTL analysis.

Population-scale genomic variation analysis based on re-sequencing has proven to be a powerful approach for elucidating genetic diversity and the genetic basis of key agronomic traits in some crops, such as rice (Yano et al., 2016), maize (Zeng et al., 2025; Liang et al., 2021), and cotton (He et al., 2021; Zhang et al., 2025). However, this powerful approach has yet to be widely applied in bitter gourd, because of the limited availability of diverse germplasm resources. Cui et al. (2020) firstly investigated the genomic variation of 187 accessions collected from 16 countries worldwide and traced the origin of large-fruited cultivated *M. charantia* var. *charantia* to the small-fruited cultivar *M. charantia* var. *muricata*, rather than to wild *M. macroloba*. To elucidate the domestication history of bitter gourd, Matsumura et al. (2020) re-sequenced 60 accessions (42 cultivars and 18 wild types) and profiled their feature by examining genetic structure, population history, genome-wide selection patterns and selection signatures of specific fruit traits, and their work provided a novel insights into the non-classical domestication genetics of bitter gourd. Based on whole genome re-sequencing of 106 bitter gourd accessions and a subsequent GWAS, Tian et al. (2023) identified *McAPRR2* as a causal gene for pericarp color. Meanwhile, genetic diversity and population structure analyses have also been performed by using simple sequence repeat (SSR) markers on panels of 51 and 96 diverse bitter gourd accessions (Alhariri et al., 2021; Mallikarjuna et al., 2023). Nevertheless, the overall progress in bitter gourd remain lags considerably behind that of other cucurbit crops.

Improving yield is a central objective for most crop breeding programs. However, genetic mapping and identifying causal gene behind yield-related traits in bitter gourd remain largely unexplored. This study therefore aims to: (i) construct a natural population consist of 180 bitter gourd accessions and use re-sequencing data disclose their genomic variation; and (ii) identify yield-associated QTL through GWAS. Anyway, we believe that our research not only provides valuable genomic information of bitter gourd but also offers significant genetic resource for improving bitter gourd yield.

## 2 Materials and methods

### 2.1 Plant materials and phenotyping for yield traits

For disclosing the genomic variation and identifying genetic resources, 180 bitter gourd germplasms were collection from 14 provinces (or regions) of China and five other countries, including Bangladesh, Vietnam, Japan, Thailand and India (**Supplementary Table 1**). All varieties were planted in 2024 at the Nanning experimental station of the Guangxi Academy of Agricultural Sciences (N: 22.83; E: 108.23). The experiment was conducted using a randomized complete block design to minimize the interference of environmental heterogeneity on experimental results. All experimental management practices followed the local conventional cultivation and management protocols, including consistent irrigation, fertilization, weeding, and pest control measures, so as to ensure that the growth environment of experimental materials was consistent with each block. For each accession, three replicate plots were set up. At the maturity stage of the materials, three representative plants were randomly selected from each plot, and at least 3 representative fruits were sampled from each selected plant for the determination of yield-related traits. Four yield-related traits were evaluated, including single fruit weight (SFW), fruit length (FL), fruit diameter (FD), and fruit flesh thickness (FFT). Finally, the average value of all the representative fruits from all plots was calculated and used as the final data for GWAS analysis.

### 2.2 Sampling, DNA isolation, and genome sequencing

A total of 180 landraces were selected for population construction and subsequent analysis. Genomic DNA was extracted with the CTAB methods. Then, 1.5 mg DNA from each sample was used to prepare sequencing library using a DirectFast DNA Library Prep Kit, following the manufacturer’s protocol. The simple process as follow: the extracted DNA was first fragmented using ultrasonic device. The fragmented DNA were then subjected to end repair, 5’phosphorylation and 3’A-tailing. Following adapter ligation, the DNA library was amplified by PCR. For sequencing on the DNBSEQ-T7 platform, for the preparation of DNA nanoballs, the double-stranded PCR products were processed into DNA nanoballs through denaturation, circularization, and enzymatic cleavage, ultimately yielding single-stranded circular DNA. The constructed library was sequenced by Agilent 2100 Bioanalyzer (Agilent Technologies, USA), and its effective concentration was precisely quantified by qPCR, with all libraries meeting the required threshold of >2 nM. Upon passing QC, sequencing was carried out on the DNBSEQ-T7 platform employing the PE150 mode.

### 2.3 Data quality checking and filtering

The remaining high-quality paired-end reads were aligned to the *Momordica charantia* L. genome (http://ftp.cngb.org) using Burrows-Wheeler Aligner (BWA) software (Li et al., 2009) with follow paremeters ‘‘mem -t 4 -k 32 -M’’. To reduce mismatches generated by PCR amplification before sequencing, duplicated reads were removed using SAMtools (v.0.1.1) (Li et al., 2009). After alignment, the genomic variants (in GVCF format for each line) were identified using the Sentieon DNASeq software package (Weber et al., 2016). Then all GVCF files of all lines were merged. To obtain high-quality variants, variants with QD > 2.0, FS < 60.0, MQ > 20.0, MQRankSum > -12.5, and ReadPosRankSum > -8.0 were retained using GATK software (Mckenna et al., 2010). With mutation detection, we obtained 6,170,339 initial variations, including 4,992,221 SNPs and 1,178,118 InDels. After filtering with following criteria: (1) two alleles only, (2) missing rate <0.2, and (4) minor allele frequency >0.01, a total of 1,775,855 polymorphic SNPs were retained. The identified SNPs and indels were further annotated with ANNOVAR software (version 2013-05-20) (Wang et al., 2010) and thus divided into the following groups on the basis of newly updated bitter melon genome annotation information: variations in intergenic regions, within 1 kb upstream (downstream) of transcription start (stop) sites, in coding sequences, and in introns.

### 2.4 Phylogenetic tree and population structure

To reduce the impact of linkage disequilibrium (LD) on population genetic analysis and ensure the independence of genetic markers, LD pruning was performed on the 1,775,855 high-quality SNPs using PLINK v1.9 software. The pruning was implemented with the parameter --indep-pairwise 50 5 0.2. After LD pruning, a total of 260,750 independent SNPs were retained to construct the individual-based neighbor joining tree based on the *p* distance using TreeBest software (v.1.9.2) (Vilella et al., 2005) with 1000 bootstrap replications. The population genetic structure was examined using ADMIXTURE (v.1.23) (Alexander et al., 2009). PCA was performed using GCTA software (Yang et al., 2011). First, we obtained the genetic relationship matrix with the parameter ‘‘–make-grm’’. Then, the top three principal components were estimated with the parameter ‘‘–pca3’’.

### 2.5 Genome-wide association study

180 accessions were used to perform GWAS using the GEMMA (genome-wide efficient mixed-model association) software package (Zhou and Stephens, 2012). For the mixed-linear-model analysis we used the following equation:


y=Xa+Sb+Km+e;

where y represents phenotype; a and b are fixed effects representing marker effects and non-marker effects, respectively; and m represents unknown random effects. X, S, and K are the incidence matrices for a, b, and m, respectively, and e is a vector of random residual effects.

Association analysis was performed using the top three principal components (PC3) as a covariate (-c) to control for population structure, and a kinship matrix (-k) derived from simple matching coefficients was included to further reduce false positives. Genetic relationships among individuals were modeled as a random effect using this K matrix, and the Wald test (-lmm 1) was applied to assess the significance of marker–phenotype associations. All analyses were conducted using the GEMMA software package with the following command line: gemma -bfile prefix -k prefix.kinship.txt -lmm 1 -n 2 -o phenotype -c prefix.pca. A total of 1,775,855 polymorphic SNPs were used for genome-wide association analysis. A genome-wide significance threshold was established using Bonferroni correction (*α* = 1), yielding a corrected threshold of -log_10_ (1/1,775,855) ≈6.25. Candidate genes were then identified by expanding the candidate region to 100 kb centered on the GWAS peak signal. Haploview (v4.2) was used to detect local linkage disequilibrium, and haplotype analysis was conducted to explore the association between haplotypes and phenotypes (Barrett et al., 2005). Further, all protein-coding genes were aligned to two integrated protein sequence databases: SwissProt (https://ftp.uniprot.org/) and NR (ftp://ftp.ncbi.nlm.nih.gov/blast/db/FASTA/nr.gz). The pathways in which the genes might be involved were assigned by BLAST against the KEGG databases (https://www.genome.jp/kegg/brite.html), with an E-value cutoff of 1e-5. Functional annotation results were merged from above strategies.

## 3 Results

### 3.1 Genomic variation and distribution

Whole-genome re-sequencing of 180 bitter gourd (*Momordica charantia*) accessions generated approximately 2.05 Tb of high-quality data. After alignment, an average sequencing depth of 33.91-fold across the accessions with approximately 99.67% of the reads mapped on reference genome, covering 96.84% of genome length (**Supplementary Table 1**). Following variant calling and filtering, we identified 1,775,855 single nucleotide polymorphisms (SNPs). Among them, 225,197 were located in upstream or downstream regions, and 1,305,972 in intergenic regions. For the remaining SNPs positioned in coding region, we annotated 37,398 non-synonymous SNPs (accounting for 2.11%), 290 splicing site variants, 152 stop-loss variants, and 947 stop-gain variants (**Table 1**). These variants are predicted to result in amino acid substitutions, transcript elongation, or premature termination of translation, respectively. Meanwhile, the overall density was 6.04 SNPs per kilobase (kb), with the highest density (11.40 SNPs/kb) observed on chromosome MC02, whereas the lowest density was on chromosome MC09 at only 4.84 SNPs/kb (**Supplementary Figure 1**, **Supplementary Table 3**). Collectively, these SNPs constitute a rich source of genetic variation for the targeted genetic improvement of key bitter gourd traits (**Supplementary Table 2**).

**Table 1:** SNPs detection and annotation statistics.

Category	Number of SNPs
Upstream	114,769
Missense	0
Stop gain	947
Stop loss	152
Synonymous	25,253
Non-synonymous	37,398
Unknown	0
Intronic	179,790
Splicing	290
UTR3	0
UTR5	0
UTR5;UTR3	0
Downstream	99,884
Upstream/downstream	10,544
Intergenic	1,305,972
ts	1,192,397
tv	583,458
ts/tv	2.043
Total	1,775,855

### 3.2 Population structure and genetic diversity analysis

**Figure 1:**
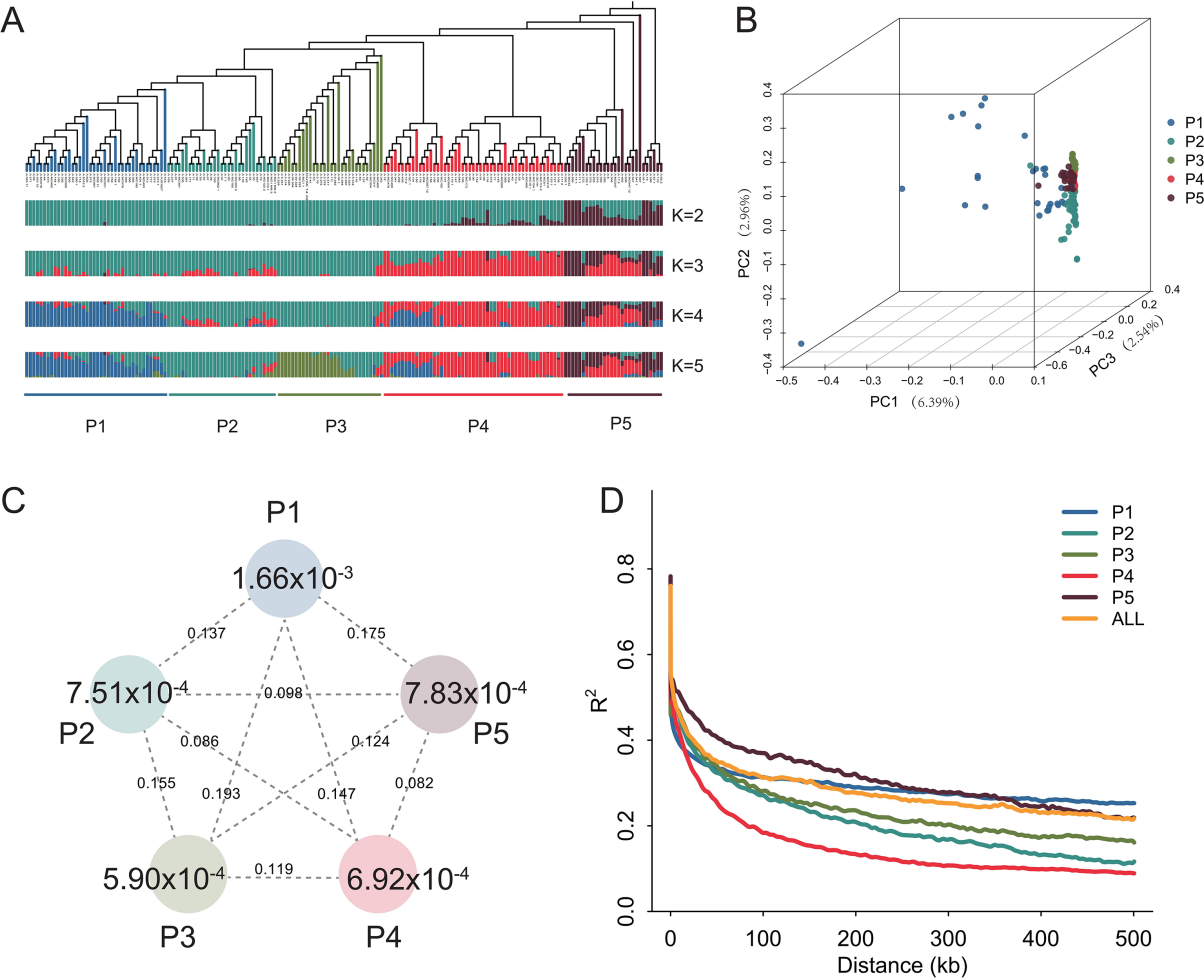
Population structure and genetic diversity analysis of the panel of 180 bitter gourd accessions. **(A)** Phylogenetic tree of 180 bitter gourd accessions and population structure based on different numbers of clusters (K = 2-5). The population is clustered into five sub-populations (P1-P5), as indicated by colored bands. Neighbor-joining tree was constructed using whole-genome SNP data. **(B)** Principal component analysis (PCA) of all bitter gourd accessions. The first three components (PC1, PC2 and PC3) are showed. **(C)** Nucleotide diversity and population differentiation across the five sub-populations. The values in the circles represents nucleotide diversity (*θπ*) for each subpopulation, and the values between the groups indicate population differentiation (*F_ST_*). **(D)** Linkage disequilibrium (LD) decay for entire population and each sub-population.

Phylogenetic analysis classified the 180 bitter gourd accessions into five distinct genetic subgroups (P1-P5), comprising by 28, 51, 30, 31, and 40 accession, respectively (**Figures 1A, B**). We further used nucleotide diversity (θπ) to evaluate genetic diversity within each subgroup. The mean θπ value for entire population was 9.03 × 10^-4^, while values ranged from 5.90 × 10^-4^ to 16.6 × 10^-4^ among the five subgroups. Notably, subgroup P1 exhibited the highest genetic diversity of 16.6 × 10^-4^ (**Figure 1C**). Despite the low genetic diversity identified in our population compared to most major crops (Zhu et al., 2026; Kang et al., 2021; Hao et al., 2020; Liu et al., 2023; Wang et al., 2021), our findings consistent with prior reports (Wang et al., 2016). In addition, linkage disequilibrium (LD) decay of the five subgroup exhibited distinct patterns (**Figure 1D**). For example, P5 showed the slowest decay (LD half = 119.32 kb), while subgroup P4 exhibited the most rapid decay (LD half = 18.14 kb). For the entire population, the LD decay half-value was 42.73 kb. This level of LD decay was higher than comparable to that reported in previous study (Cui et al., 2020). To identify genomic regions under putative selection, we performed a comparative analysis of nucleotide diversity (π) and fixation index (F*_st_*) across subgroups. We identified 15,049 genomic regions with significant diversity reduction (top 5% of π ratios), and 7,429 regions showing significant divergence (top 5% of F_st_ values). Integrating both π and F_st_ signals revealed 1,143 overlapping genomic regions likely under putative selection, spanning approximately 16.84 Mb (**Supplementary Table 4**).

### 3.3 Phenotypic variation across accessions

**Figure 2:**
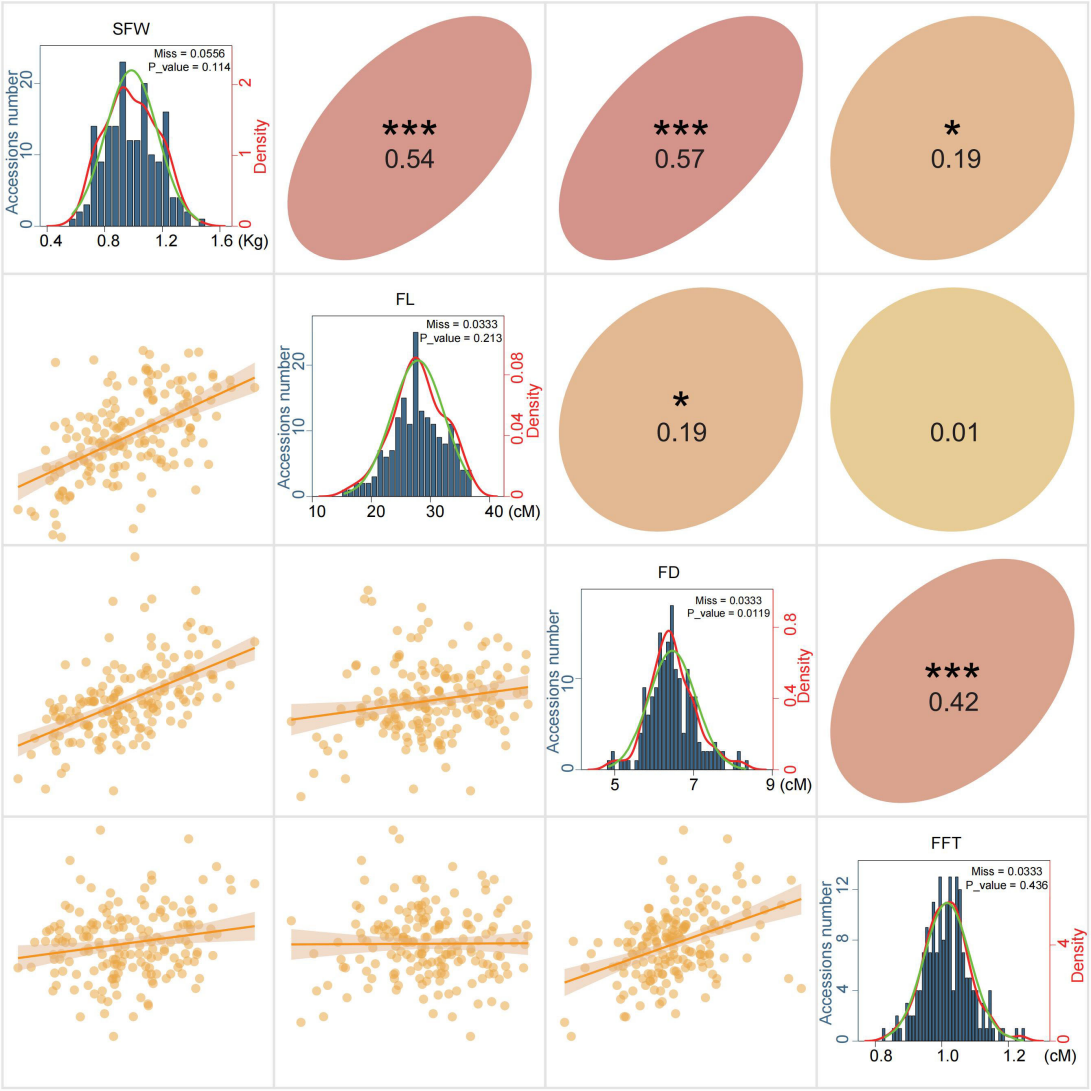
Correlations among four yield-related traits in the panel of 180 bitter gourd accessions. SFW, Single fruit length; FL, Fruit length; FD, Fruit diameter; FFT, Fruit flesh thickness. The *, **, and *** represent significant correlations, with p-values less than 0.05, 0.01, and 0.001, respectively.

To facilitate molecular breeding, we investigated four yield-related traits of the above bitter gourd population, including SFW, FL, FD and FFT (**Supplementary Table 5**). For these traits, the coefficient of variation (CV) for these traits ranged from 6.833% to 18.379% (**Table 2**). As most absolute values of kurtosis and skewness values were below 1, suggesting that all the observed traits approximately fitted normal frequency distributions, further normality test confirmed that three traits exhibited a perfect fit for the normal distribution (*p >*0.1), whereas FD exhibited slightly skewed distributions (**Table 2**). Correlation analysis revealed that SFW had strong positive correlations with FL (r = 0.54, *p* < 0.001) and FD (*r* = 0.57, *p* < 0.001). In contrast, its correlation with FFT was weak but still significant (r = 0.19, p < 0.05) (**Figure 2**). These phenotypic correlations indicated SFW, FL and FD may share some common genetic base, which critical for improving yield through molecular breeding.

**Table 2:** Phenotypic variation for the panel of 180 bitter gourd accessions.

Traits	Mean	SD	CV%	Min	Max	Skew	Kurt	Normality test (*P >*0.1)
SFW	0.493	0.091	18.379	0.288	0.730	0.106	-0.673	0.114
FL	27.890	4.461	15.993	15.475	36.963	-0.202	-0.210	0.213
FD	6.461	0.598	9.250	4.855	8.314	0.373	0.861	0.012
FFT	1.016	0.069	6.833	0.830	1.244	0.272	0.582	0.436

### 3.4 Genome-wide association analysis and predicted candidate genes for yield-related traits

**Figure 3:**
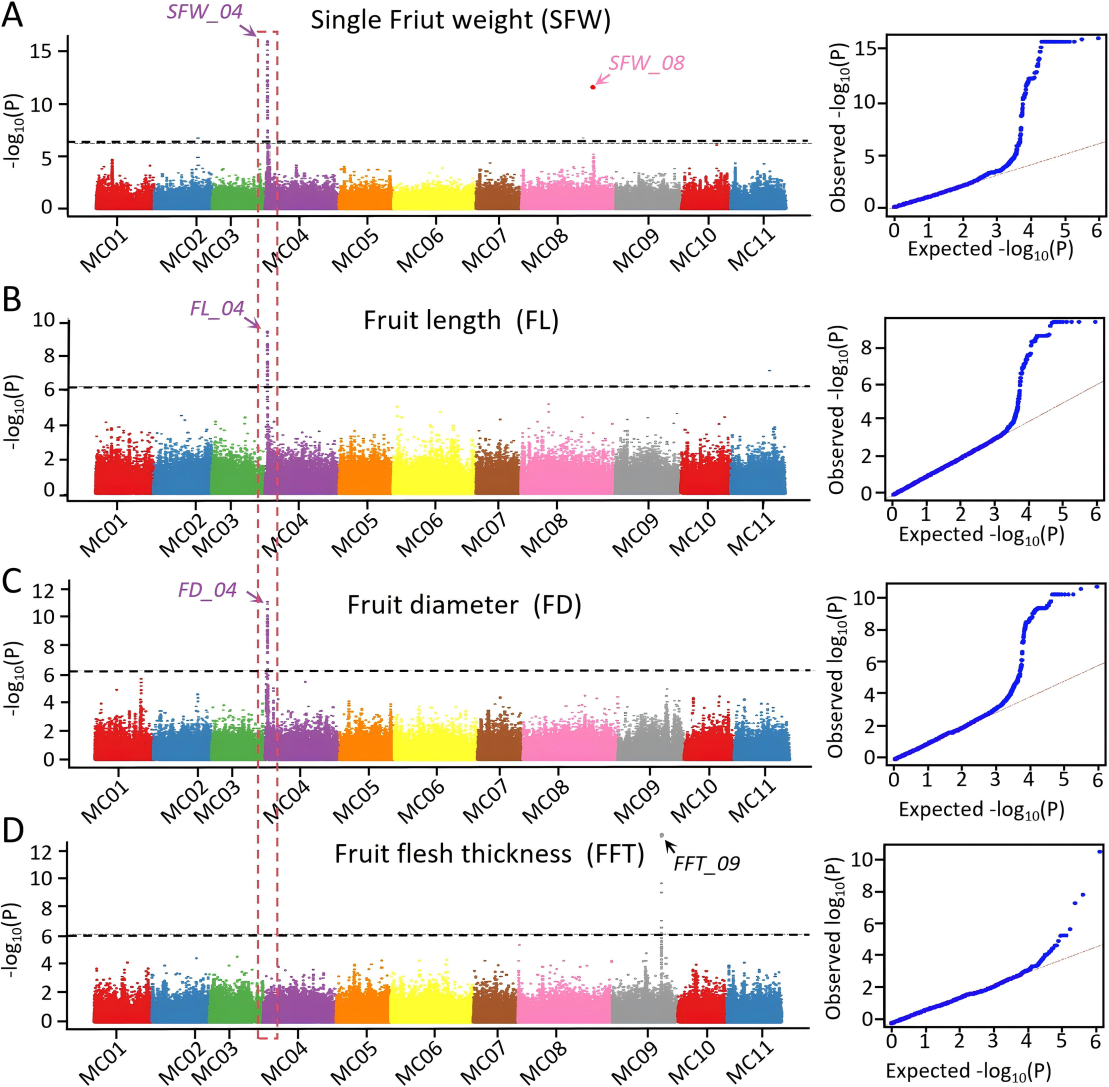
Genome-wide association analysis for yield-related traits. Manhattan and QQ plot of GWAS for **(A)** Single fruit weight, SFW; **(B)** Fruit length, FL; **(C)** Fruit diameter, FD; **(D)** Fruit flesh thickness, FFT. Dashed line indicates the significance threshold (-log_10_*P* = 6.25).

Genome-wide association analysis of four yield-related traits identified five significant loci distributed across chromosomes MC04, MC08, and MC09 (**Figure 3**, **Supplementary Table 6**). A major pleiotropic locus was detected on MC04, which simultaneously associated with SFW, FL, and FD. This locus explained approximately 16.69%, 11.96% and 8.10% of the phenotypic variance for SFW, FL, and FD, with association signals reaching −log10(P) values of 15.92, 9.43, and 11.04, respectively. In addition, two other trait-specific loci were identified. On MC08, a significant association for SFW was observed, accounting for 20.80% of the phenotypic variance and a −log10(P) value of 11.56. Similarly, one locus on MC09 was associated with FFT, explaining 29.51% of its phenotypic variation and exhibiting a −log10(P) value of 13.08. Collectively, these results highlight both pleiotropic and trait-specific genetic influences underlying the variation in yield-related traits. Further, LD block analysis suggested that the pleiotropic locus (i.e. SFW_04, FL_04 and FD_04) was localized to the interval of 0.836-0.952Mb on chromosome MC04. Analysis of the candidate interval identified a total of 14 annotated genes. Among these, four genes were found to carry SNP variants within their exon regions, all of which result in non-synonymous amino acid changes. These genes include: MC04g0117 (a hypothetical protein with a BRCA1-associated domain), MC04g0120 (a trehalose-phosphate synthase), MC04g0121 (an ATP-dependent zinc metalloprotease, FTSH 2), and MC04g0126 (a putative protein phosphatase 2C). Two trait-specific loci, SFW_08 and FFT_09, annotating one and two candidate genes with potential functional impact, respectively. Within the SFW_08 locus, the gene MC08g1837 was prioritized, which encodes a 2-hydroxyacyl-CoA lyase. A nonsynonymous mutation within its exonic region suggests a possible alteration in enzyme activity. At the FFT_09 locus, our analysis revealed two compelling candidates: the annotated tyrosine/DOPA decarboxylase gene MC09g1204 (K01592, EC 4.1.1.25) and a newly annotated paralog, MC09g_new0383, predicted to share the same enzymatic function. Both genes contain nonsynonymous variants in their exons, pointing to potential structural or functional modifications of this key enzyme involved in aromatic amino acid decarboxylation and secondary metabolite synthesis. These nonsynonymous exonic variants represent strong candidate causative mutations that are predicted to alter protein function, thereby possibly contributing to the observed phenotypic variation.

### 3.5 Haplotypes analysis reveals phenotypic effects in natural populations

**Figure 4:**
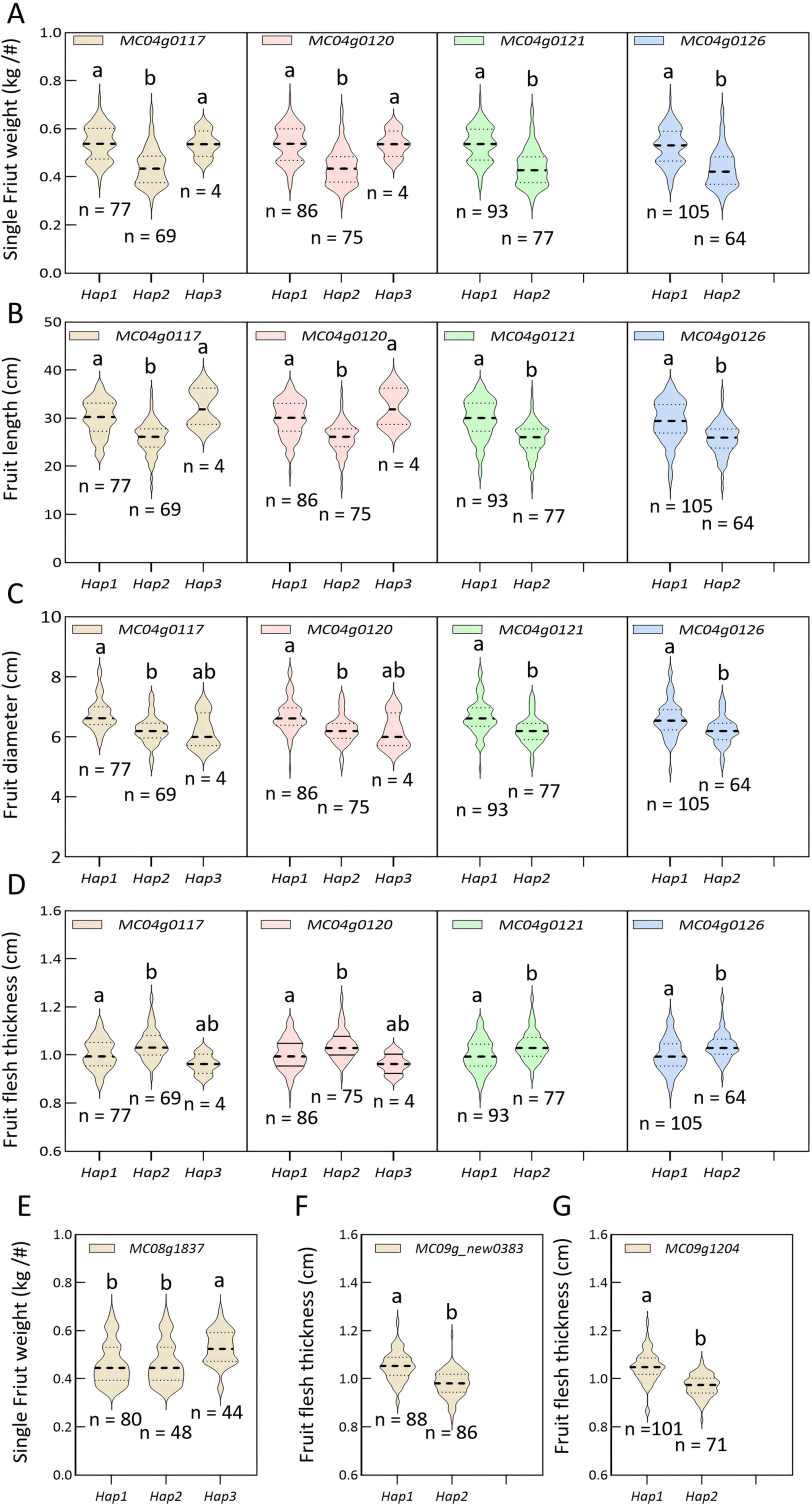
Haplotype analysis of candidate genes associated with yield-related traits in bitter gourd. Phenotypic effects of haplotypes from four candidate genes (MC04g0117, MC04g0120, MC04g0121, and MC04g0126) within a pleiotropic locus on four distinct traits: single fruit weight, SFW **(A)**, fruit length, FL **(B)**, fruit diameter, FD **(C)**, and fruit flesh thickness, FFT **(D)**. Phenotypic effect of haplotypes of the candidate gene MC08g1837 on SFW **(E)**. Phenotypic effects of haplotypes from two candidate genes, MC09g_new0383 **(F)** and MC09g1204 **(G)**, on FFT. Data are presented as mean ± standard error (SE). Different lowercase letters above the bars indicate statistically significant differences among haplotypes based on one-way ANOVA followed by Tukey’s HSD test (*p* < 0.05).

Haplotype-based association analysis was performed to assess the genetic effects of candidate genes in a natural population (**Figure 4**). For the pleiotropic loci, genes MC04g0117 and MC04g0120 each exhibited three haplotypes, with Hap3 representing a rare variant present in only four accessions (**Supplementary Figures 2**, **3**). In contrast, MC04g0121 and MC04g0126 each displayed two major haplotypes (**Supplementary Figures 4**, **5**). Compared with Hap2, Hap1 in all four genes significantly increased SFW by 20.46%–22.68%, FL by 13.68%–16.09%, and FD by 6.28%–8.44% (**Figures 4 A–D**). Although a significant difference in SFW was observed between Hap3 and Hap1 for MC04g0117 and MC04g0120, this result may be attributed to the limited sample size of Hap3. Interestingly, while the initial GWAS did not detect an association between this pleiotropic region and FFT, haplotype analysis revealed that Hap2 consistently increased FFT by 4.00%–4.31% compared with Hap1 across all four candidates, suggesting a previously undetected role in this trait (**Figures 4A–D**). These findings indicate that one or more of these genes play key regulatory roles, though the precise mechanisms require further validation. Meanwhile, for the SFW_08 locus, candidate gene MC08g1837 showed three haplotypes (**Supplementary Figure 6**). Hap2 significantly increased SFW by 9.42% and 14.33% compared with Hap1 and Hap3, respectively, though no significant difference was observed between Hap2 and Hap3 (**Figure 4E**). At the FFT_09 locus, two candidate genes, MC09g_new0383 and MC09g1204, each formed two haplotypes, with Hap1 increasing FFT by 7.57% and 8.18% compared with Hap2 (**Figures 4F, G**, **Supplementary Figures 7**, **8**).

Collectively, these haplotype analyses underscore the functional relevance of the candidate genes and reveal haplotype-specific effects on agronomic traits, including previously undetected contributions to FFT. However, it should be also acknowledged that the small sample size of Hap3 (n =4) may reduce the statistical reliability of the observed phenotypic differences between Hap3 and other haplotypes, which is a limitation of the present study.

## 4 Discussion

The utilization of single nucleotide polymorphism (SNP) markers for genotyping represents a milestone in modern crop genetics, offering superior polymorphism and resolution over traditional marker systems such as SSRs and AFLPs. Advances in high-throughput sequencing, particularly whole-genome resequencing, have enabled the comprehensive acquisition of SNP information, profoundly enhancing our understanding of genetic diversity, evolutionary history, and domestication processes in plants (Xu and Bai, 2015). GWAS effectively integrate these high-density SNP datasets with phenotypic information to dissect the genetic architecture of complex traits, significantly accelerating the pace of crop improvement. The broad utility of this approach is evidenced by its successful application in over 180 plant species, leading to the identification of thousands of trait-associated loci (Song et al., 2023).

Bitter gourd (*Momordica charantia* L.) holds considerable economic and medicinal value globally, yet its genetic research has progressed more slowly than that of major staple crops. This lag is primarily attributable to historically limited genomic resources and germplasm collections. To date, only a limited number of population-genetic studies have been conducted for this species (Cui et al., 2020; Matsumura et al., 2020). Our study directly addresses this resource gap by performing whole-genome resequencing on a diverse panel of 180 accessions. This effort constitutes the second-largest genomic dataset for bitter gourd to date and yielded a robust resource of 1,775,855 high-quality SNPs. This dense SNP map provides an unprecedented resolution for analyzing genomic variation and deciphering population structure within cultivated bitter gourd. Meanwhile, we delineated the 180 accessions into five distinct genetic subpopulations (P1—P5), revealing clear substructure and varying levels of diversity. Notably, subpopulation P1 exhibited the highest nucleotide diversity (θπ = 16.6 × 10^-4^). However, the overall genetic diversity across the panel remains relatively low when compared to other cucurbit crops. This finding corroborates the hypothesis that bitter gourd underwent significant genetic bottlenecks during its domestication and breeding history. These findings highlight the necessity to broaden the genetic base of bitter gourd in future breeding, for instance by introducing wild relatives and landraces to enhance genetic variation.

The observed strong positive correlations between SFW and both FL and FD, coupled with the identification of a major pleiotropic locus on chromosome MC04 simultaneously associated with these three traits, provide compelling genetic evidence for their coordinated contribution to yield in bitter gourd. The high phenotypic correlations (r > 0.54) suggest that FL and FD are primary morphological components directly constituting SFW. The co-localization of significant GWAS signals for SFW, FL, and FD within the same genomic region (0.836-0.952 Mb on MC04) strongly implies that this locus harbors genetic variant(s) with pleiotropic effects, potentially regulating a common developmental pathway influencing overall fruit size and expansion. This genetic architecture is advantageous for molecular breeding, as selection for favorable alleles at this locus could simultaneously enhance multiple yield components. In contrast, the weaker correlation and independent genetic locus (on MC09) for fruit flesh thickness (FFT) indicate that this trait is governed by a more distinct set of genes, contributing to SFW variation in a less direct or additive manner, possibly through influences on fruit density or internal structure.

The haplotype analysis revealed that candidate genes within the pleiotropic locus (e.g., MC04g0117, MC04g0120, MC04g0121, MC04g0126) not only affected the three primary traits (SFW, FL, FD) as identified by GWAS but also showed significant haplotype effects on FFT, a trait not originally associated with this region in the GWAS. This apparent discrepancy can likely be attributed to the genetic resolution differences between the two methods and the influence of Linkage Disequilibrium (LD). GWAS typically detects associations based on single-marker statistics, which may lack power for traits with complex or subtler genetic effects. Haplotype analysis, by integrating information from multiple linked variants, can capture the combined effect of a genomic block more effectively. The observed haplotype effects on FFT suggest that the causal variant(s) influencing this trait might be in strong LD with the SNPs/haplotypes tagged in the analysis, but its individual effect size may have been below the stringent GWAS detection threshold. Alternatively, the haplotype might be tagging a cis-regulatory variant that moderately modulates gene expression, affecting FFT subtly while exerting stronger effects on fruit size. The LD block structure likely groups multiple potentially functional polymorphisms (including those in the four candidate genes) into a limited number of haplotypes, making it challenging to pinpoint a single causal gene or variant for each specific trait. This highlights the complex genetic interplay within this pleiotropic region, where one or several closely linked genes may have multifaceted roles in regulating various aspects of fruit development, including size and flesh thickness. Further fine-mapping and functional validation are required to dissect whether the observed multi-trait effects stem from a single gene with true pleiotropy or multiple tightly linked genes each affecting different traits.

Strong linkage disequilibrium (LD) in the MC04 locus complicates the distinction among the four identified candidate genes (MC04g0117, MC04g0120, MC04g0121, MC04g0126), as tight LD may lead to hitchhiking effects. Among them, MC04g0120 (trehalose-phosphate synthase, TPS) and MC04g0126 (protein phosphatase 2C, PP2C) are biologically plausible regulators of fruit development, supported by studies on their orthologs in cucurbits: TPS genes regulate fruit expansion and sugar accumulation in watermelon and cucumber, while PP2C genes modulate ABA signaling and cell expansion related to fruit size in pumpkin (Fu et al., 2023; Yuan et al., 2022; Guo et al., 2021). In contrast, MC04g0117 (BRCA1-associated domain-containing hypothetical protein) and MC04g0121 (FTSH 2 metalloprotease) lack direct evidence linking them to fruit development, making them likely LD-linked candidates rather than causal regulators. To validate causal variants, additional gene expression data (e.g., stage-specific expression during fruit development) and high-resolution fine-mapping are needed, which aligns with GWAS best practices for prioritizing functional candidates and resolving LD block-related ambiguities.

## 5 Conclusions

This study conducted whole-genome resequencing of 180 bitter gourd accessions, constructing a high-density variation map and analyzing population structure. We identified a major pleiotropic locus on chromosome MC04 harboring candidate genes MC04g0117, MC04g0120, MC04g0121, and MC04g0126, which governing key fruit size traits. While these genes provide prime targets for molecular breeding, the extensive LD complicates causal gene assignment. Future fine-mapping and functional studies are essential to dissect this locus and validate the true genetic drivers of these complex traits.

## Data Availability

The data in the public repositories include all raw reads for bitter gourd genomics in the BlG Data Center under BioProject ID PRJCA056831.

## Supplementary material

Supplementary Figure 1 Heatmap of SNP distribution on each chromosome of bitter gourd. SNP density are shown in 1 Mb windows sliding 1000 kb.

Supplementary Figure 2 Genetic Variation and haplotype distribution of MCO4g0117.

Supplementary Figure 3 Genetic Variation and haplotype distribution of MC04g0120.

Supplementary Figure 4 Genetic Variation and haplotype distribution of MC04g0121.

Supplementary Figure 5 Genetic Variation and haplotype distribution of MC04g0126.

Supplementary Figure 6 Genetic Variation and haplotype distribution of MC08g1837.

Supplementary Figure 7 Genetic Variation and haplotype distribution of MC09g_new0383.

Supplementary Figure 8 Genetic Variation and haplotype distribution of MC09g1204.
