# Supplementary figures and images for: Re-sequencing of 180 bitter gourd accessions uncovering the genetic basis of key horticultural traits
Source: Front Plant Sci. 2026 Apr 16;17:1807943. doi: 10.3389/fpls.2026.1807943 (PMC13128658; doi:10.3389/fpls.2026.1807943)

# The number of SNPs within 1Mb window size

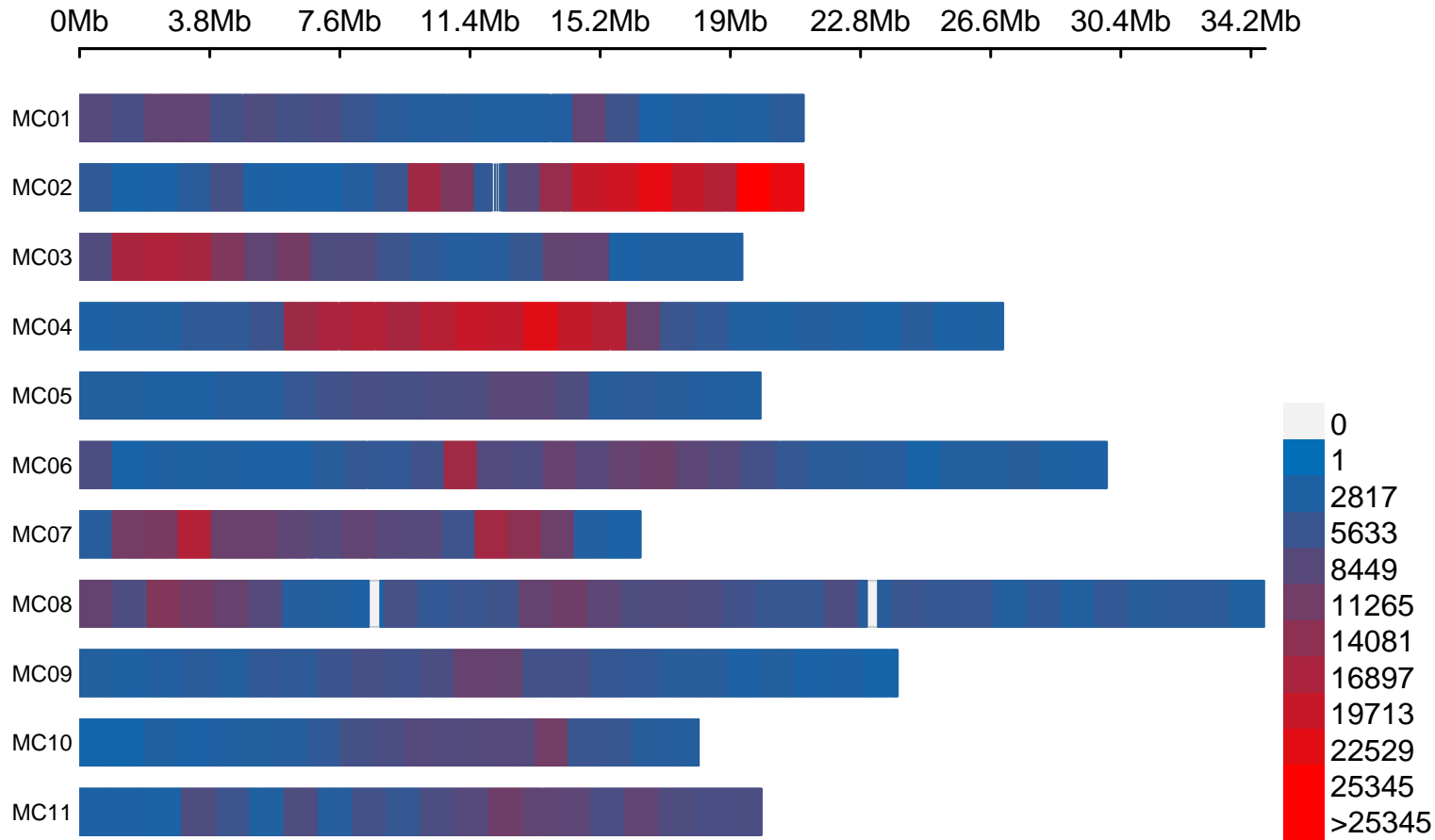

Supplement: Supplementary Figure 1 — Heatmap of SNP distribution on each chromosome of bitter gourd. SNP density are shown in 1 Mb windows sliding 1000 kb. [file DataSheet1.pdf]

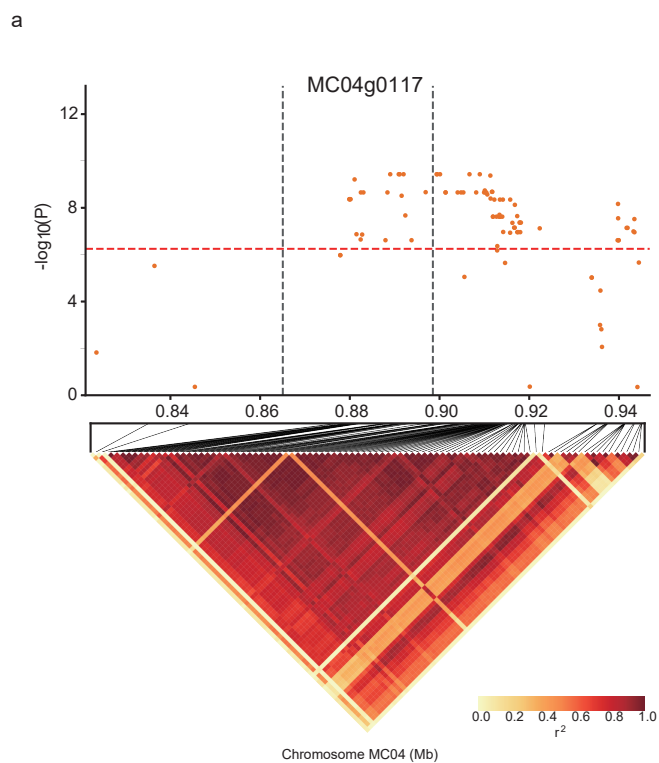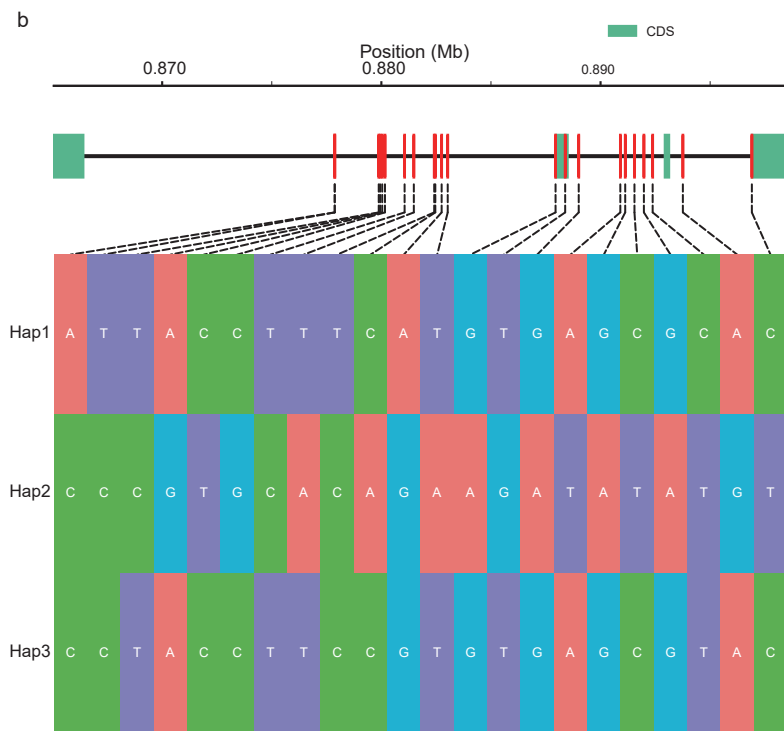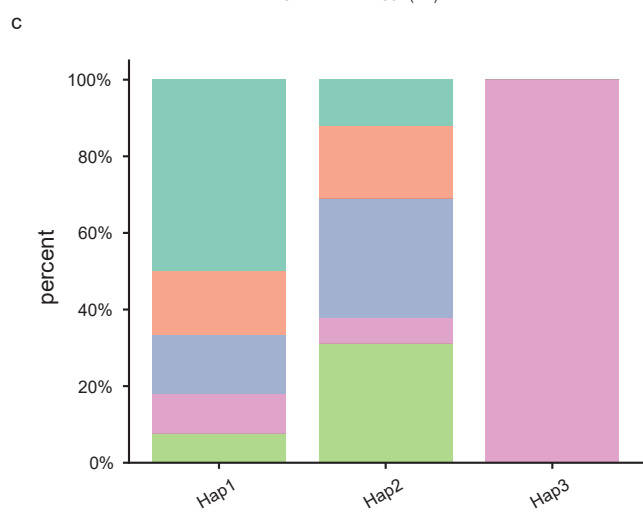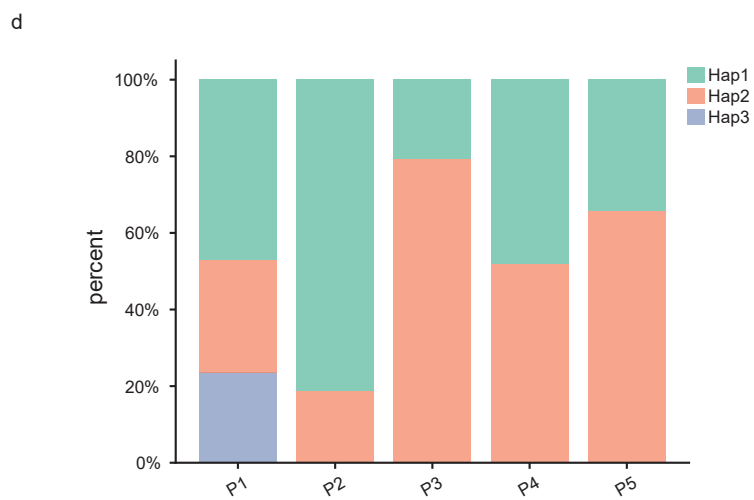

Supplement: Supplementary Figure 2 — Genetic Variation and haplotype distribution of MCO4g0117. [file DataSheet2.pdf]

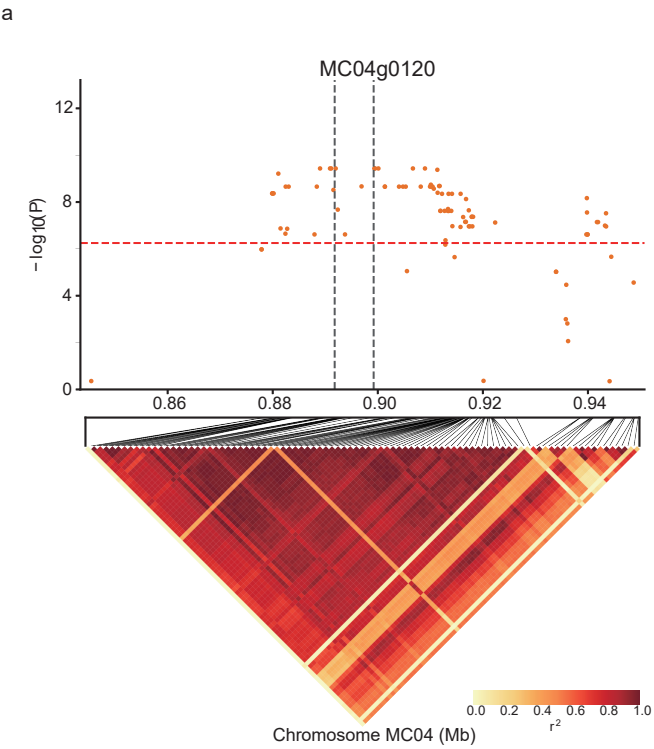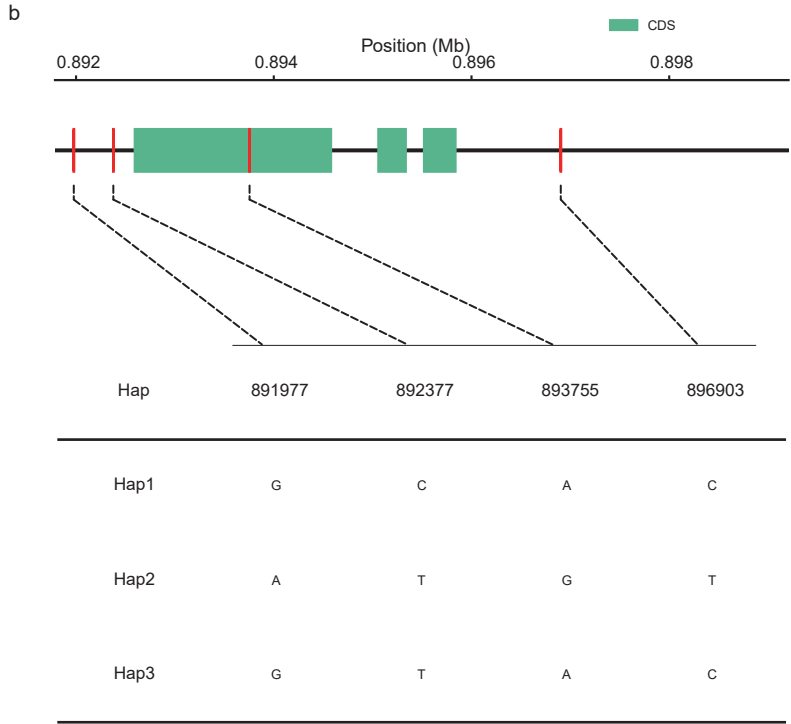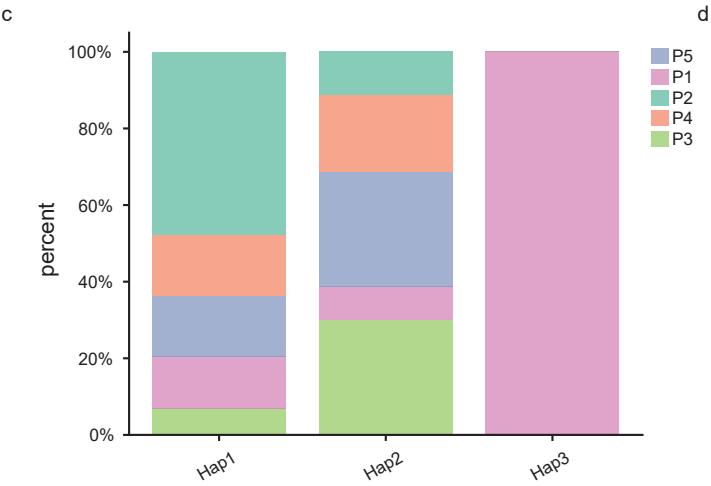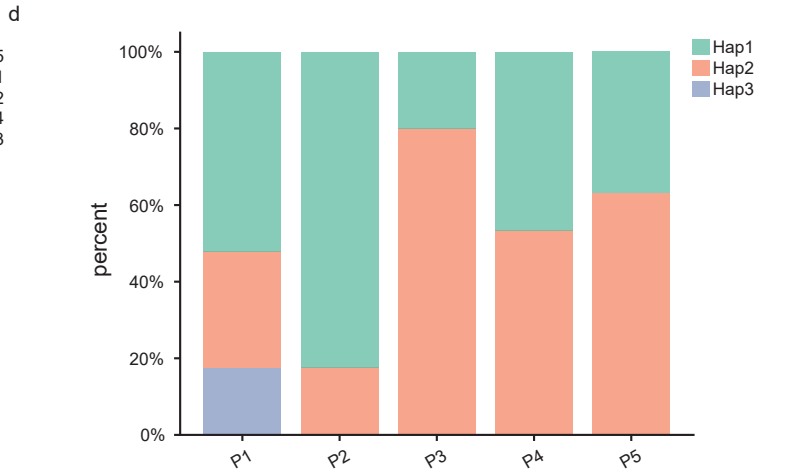

Supplement: Supplementary Figure 3 — Genetic Variation and haplotype distribution of MC04g0120. [file DataSheet3.pdf]

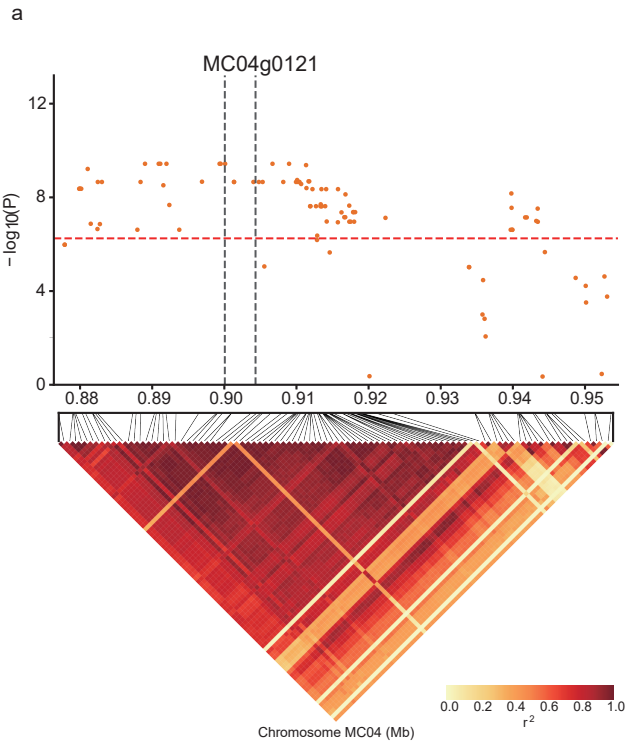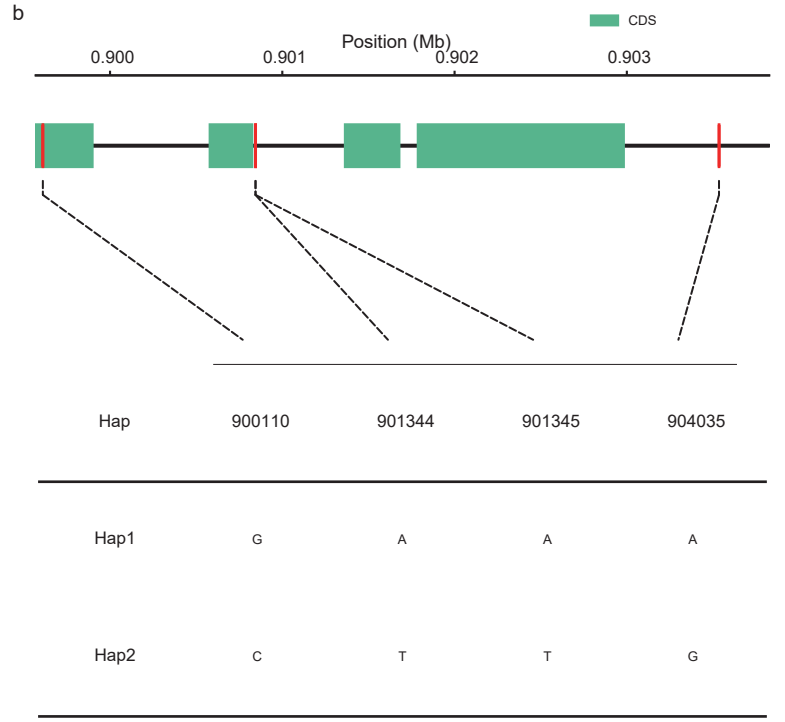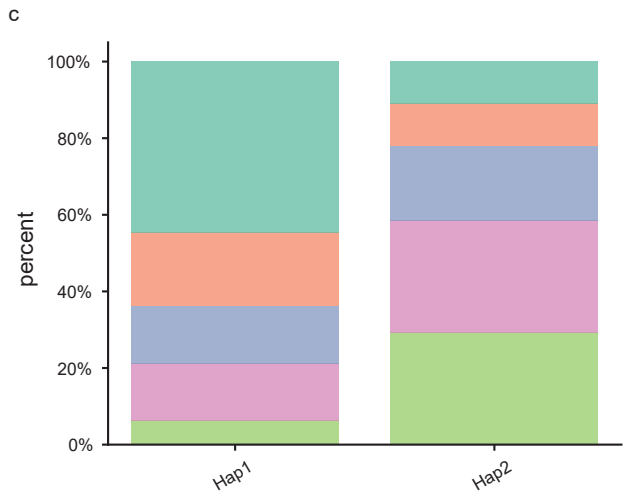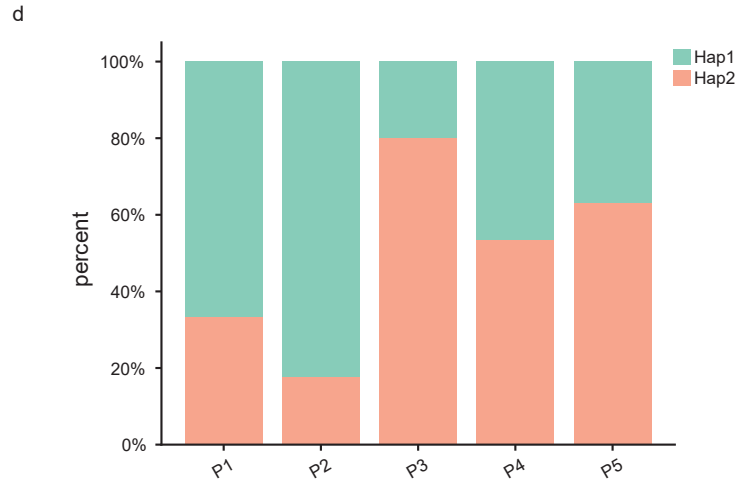

Supplement: Supplementary Figure 4 — Genetic Variation and haplotype distribution of MC04g0121. [file DataSheet4.pdf]

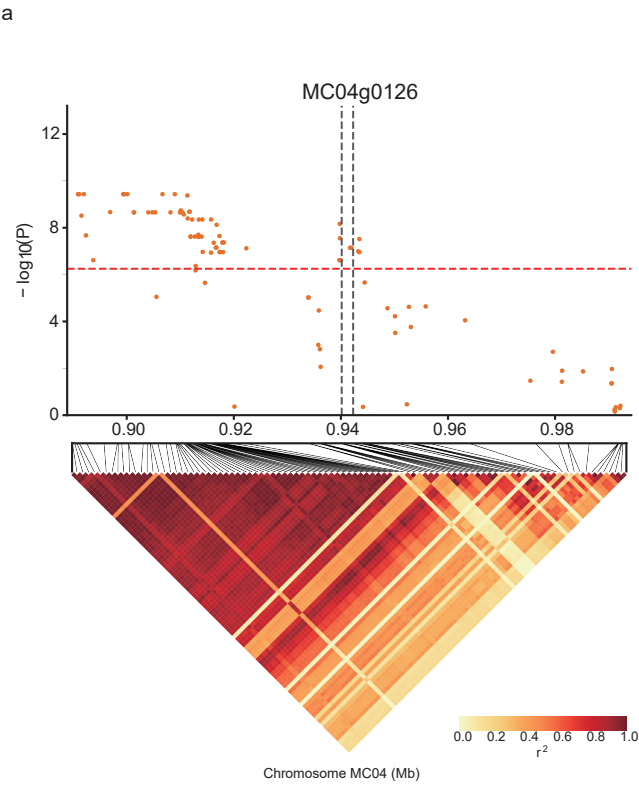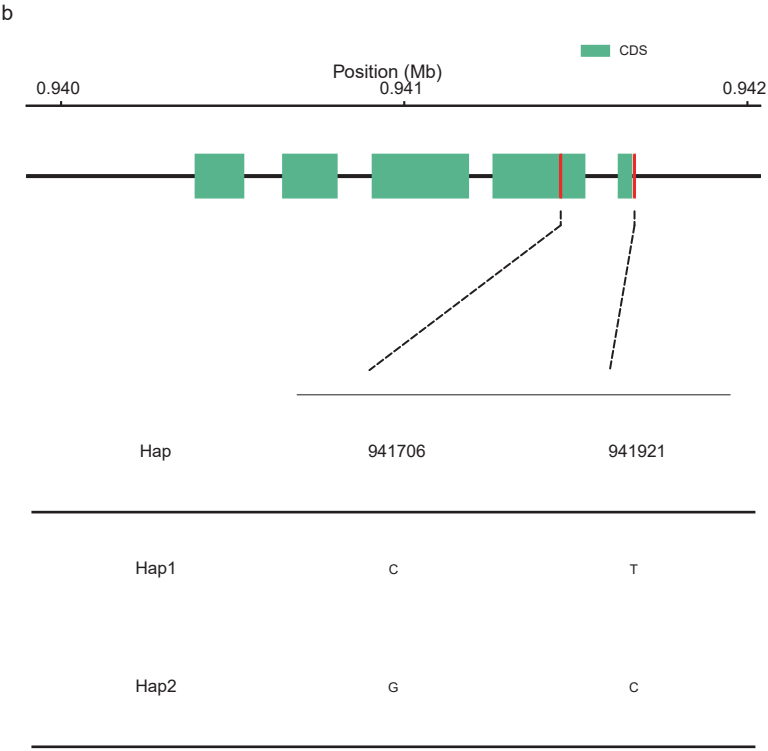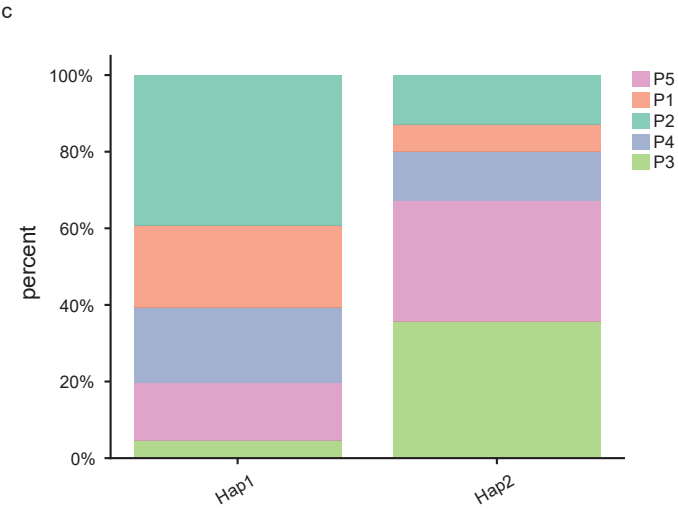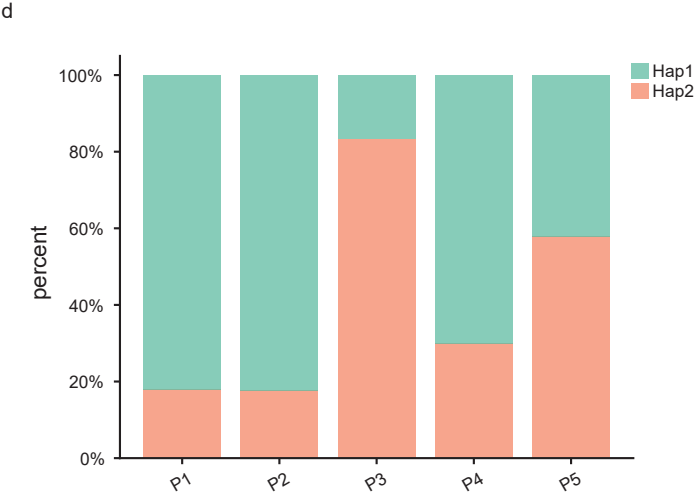

Supplement: Supplementary Figure 5 — Genetic Variation and haplotype distribution of MC04g0126. [file DataSheet5.pdf]

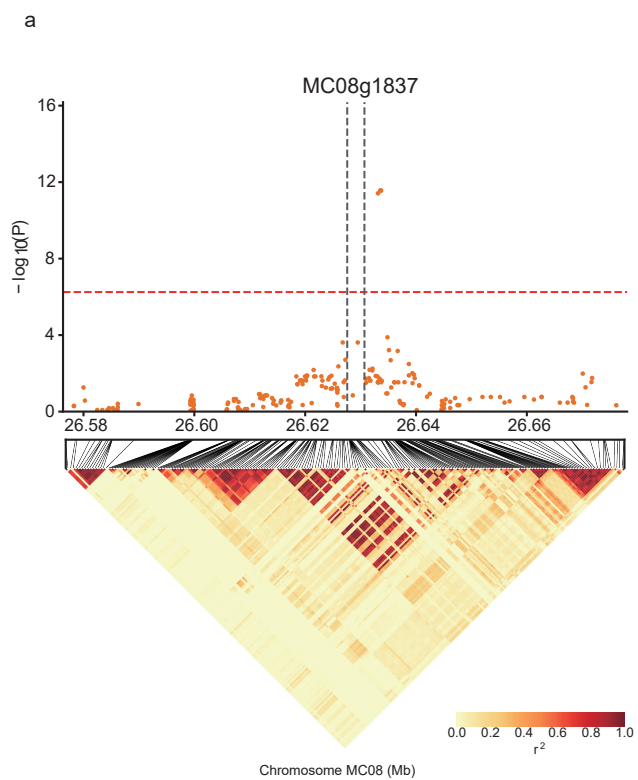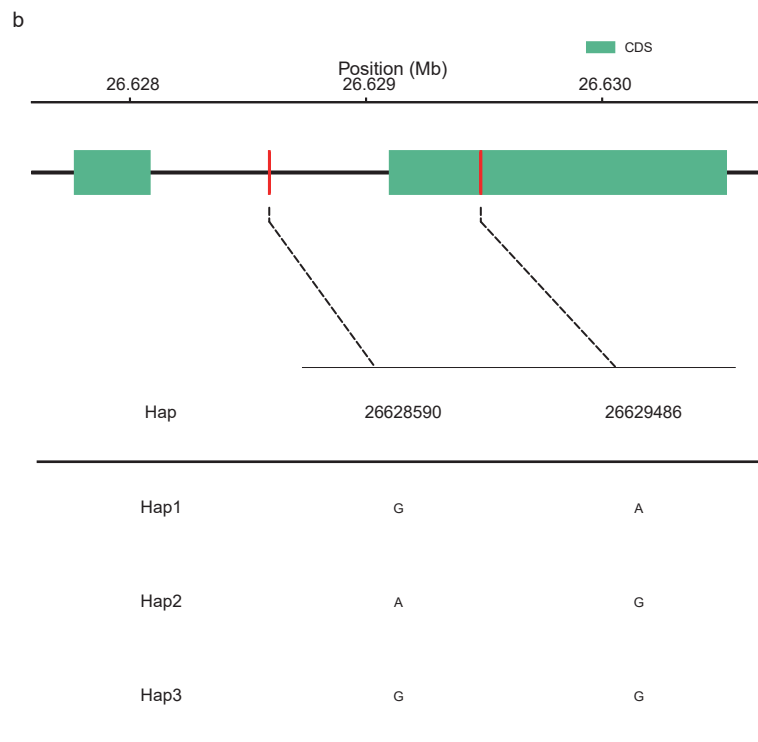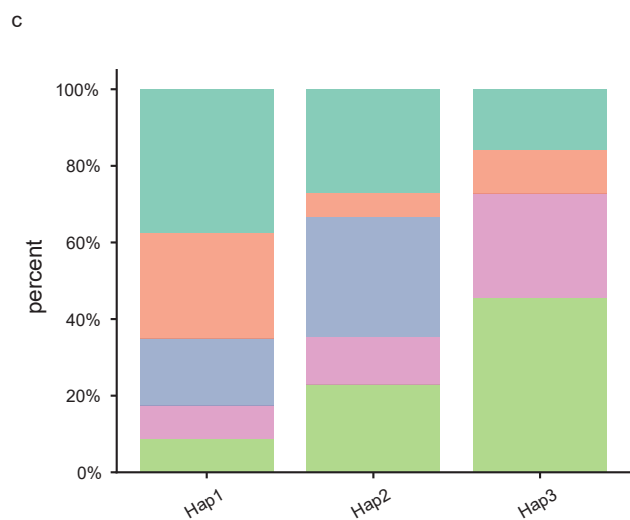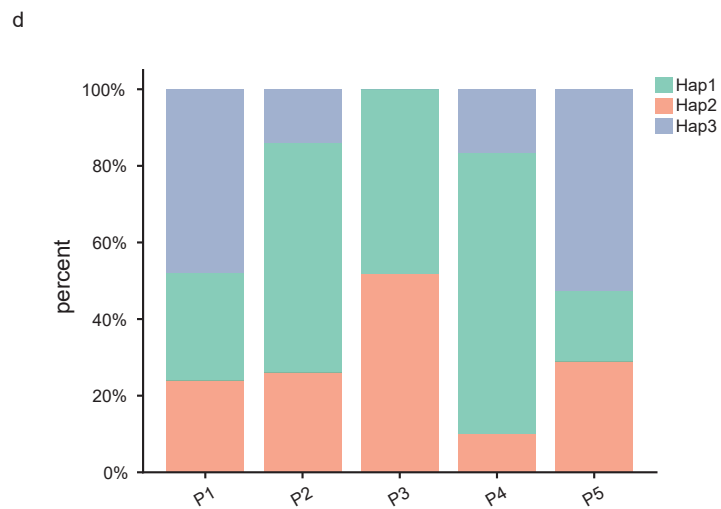

Supplement: Supplementary Figure 6 — Genetic Variation and haplotype distribution of MC08g1837. [file DataSheet6.pdf]

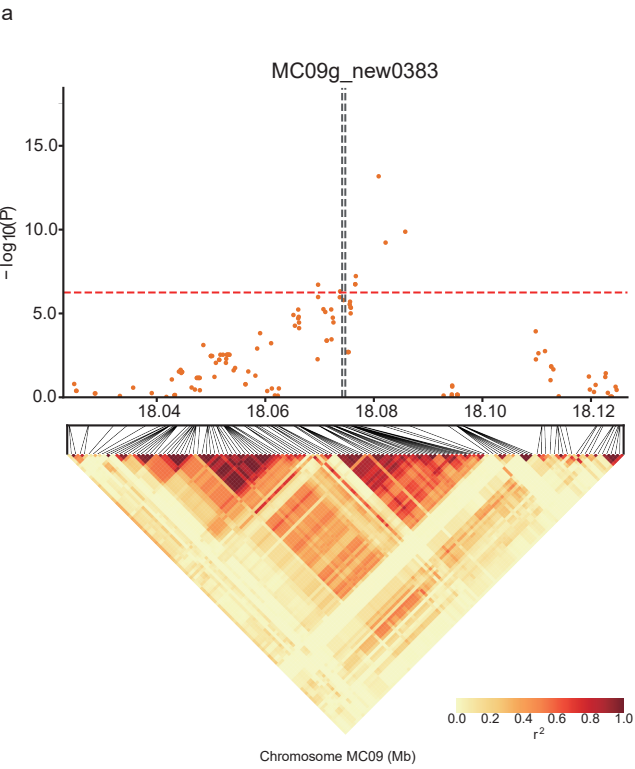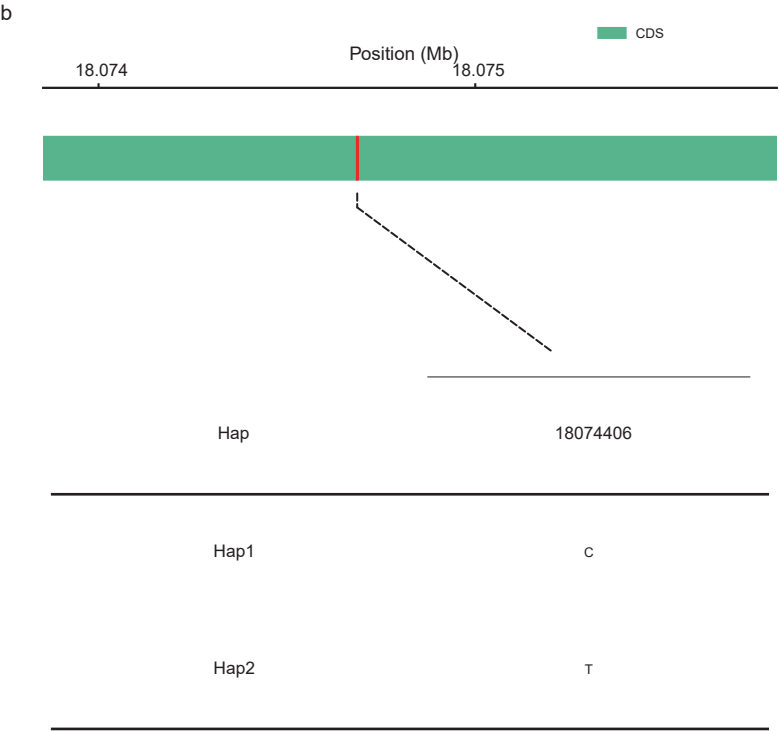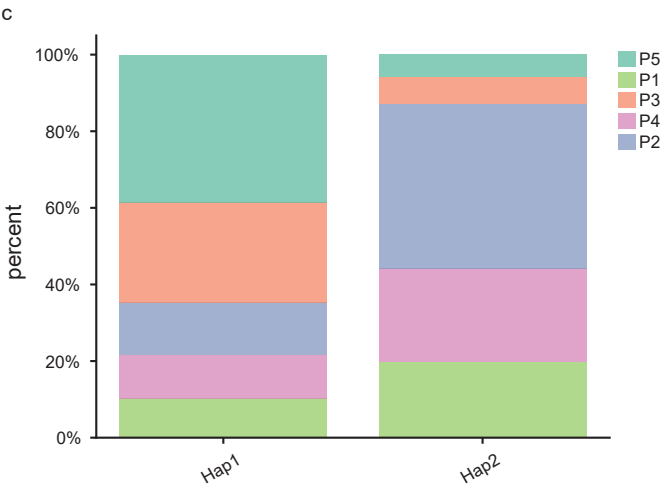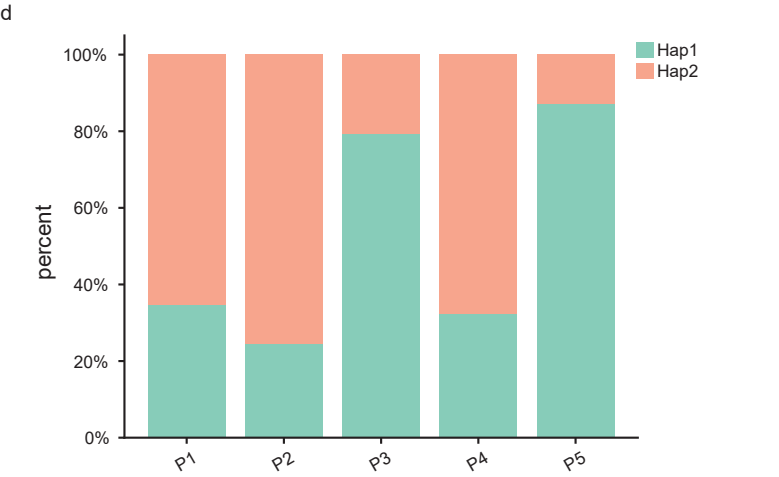

Supplement: Supplementary Figure 7 — Genetic Variation and haplotype distribution of MC09g_new0383. [file DataSheet7.pdf]

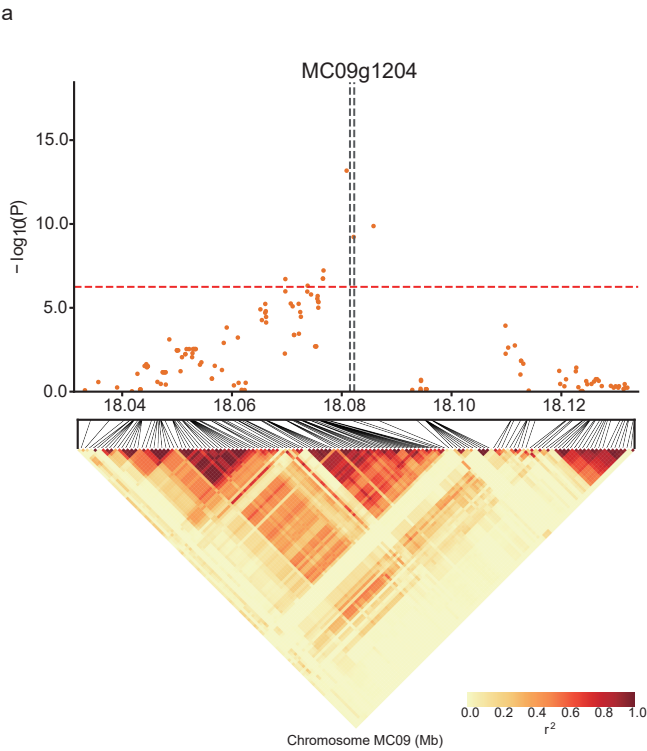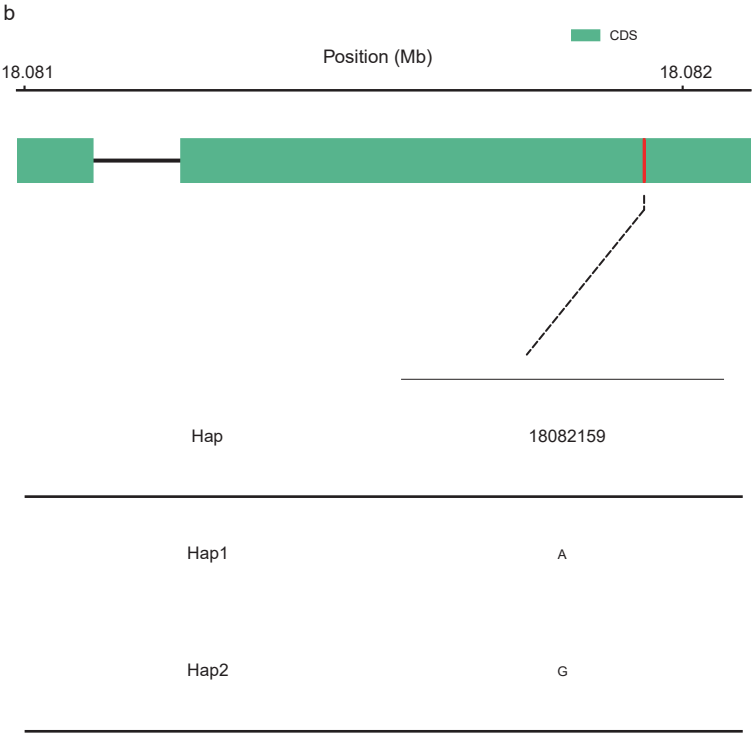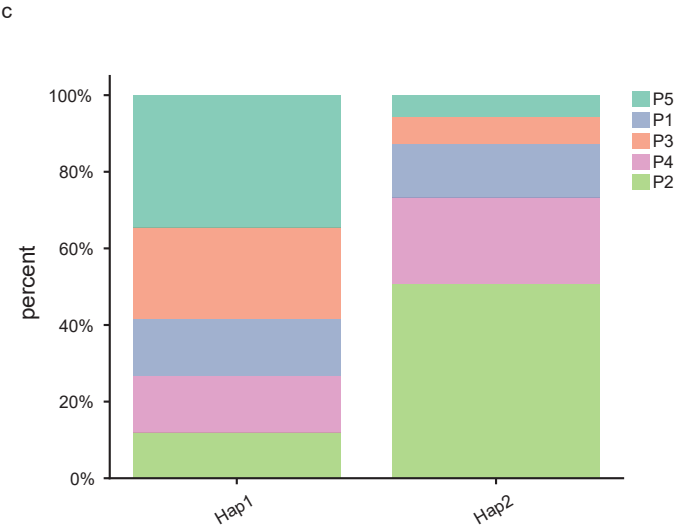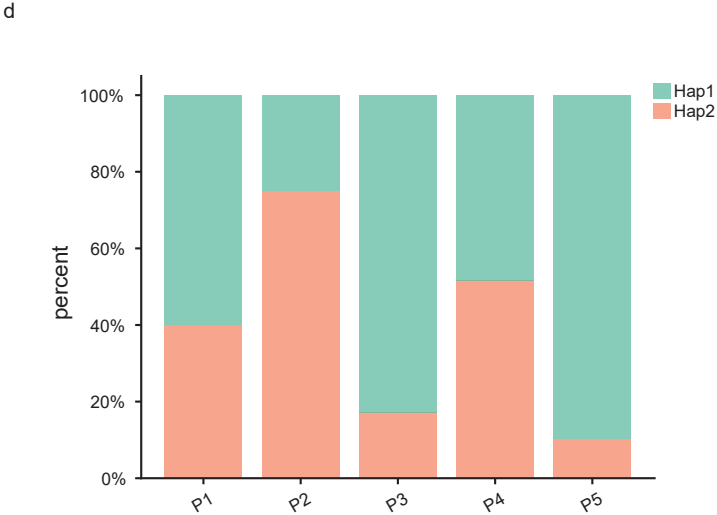

Supplement: Supplementary Figure 8 — Genetic Variation and haplotype distribution of MC09g1204. [file DataSheet8.pdf]
